# Supplementary material for: The study strategies of small liberal arts college students before and after COVID-19
Source: PLoS One. 2022 Dec 8;17(12):e0278666. doi: 10.1371/journal.pone.0278666 (PMC9731427; doi:10.1371/journal.pone.0278666)
Supplement: S1 File — S1 Table compares the current results to three prior examinations of student study strategies. S2 Table provides a detailed analysis of student study strategies before and after COVID-19. (DOCX) [file pone.0278666.s001.docx]

Table S1

*Comparison of current results to prior research on student study strategies.*

| Questions | Responses | Kornell & Bjork (2007) | Hartwig & Dunlosky (2012) | Morehead et al. (2016) | Present study |
| --- | --- | --- | --- | --- | --- |
| 1.Would you study the way you do because a teacher (or teachers) taught you to study that way? | Yes | 20% | 36% | 36% | 52% |
|  | No | 80% | 64% | 64% | 47% |
| 2. How do you decide what to study next? | Whatever's due soonest/overdue | 59% | 56% | 63% | 62% |
|  | Whatever I haven't studied for the longest time | 4% | 5% | 3% | 1% |
|  | Whatever I find interesting. | 4% | 2% | 4% | 7% |
|  | Whatever I feel I'm doing the worst in | 22% | 24% | 9% | 8% |
|  | I plan my study schedule ahead of time, and I study whatever I've scheduled. | 11% | 13% | 21% | 22% |
| 3. Do you usually return to course material to review it after a course has ended? | Yes | 14% | 23% | 28% | 23% |
|  | No | 86% | 78% | 72% | 76% |
| 4. All other things being equal, what do you study more for? | Essay/short answer exams | 29% | 20% | 27% | 25% |
|  | Multiple choice exams | 22% | 22% | 22% | 21% |
|  | About the same | 49% | 58% | 51% | 53% |
| 5. When you study, do you typically read a textbook/article more than once? | Yes, I reread whole chapter/articles | 16% | 19% | 17% | 11% |
|  | I reread sections that I underlined highlighted or marked | 60% | 64% | 58% | 61% |
|  | Not usually | 23% | 17% | 25% | 28% |
| 6. If you quiz yourself while you study (either using a quiz at the end of a chapter, or a practice quiz, or flashcards, or something else) why do you do it? | I learn more that way that I would through rereading | 18% | 27% | 31% | 30% |
|  | To figure out how well I have learned the information I'm studying | 68% | 54% | 49% | 48% |
|  | I find quizzing more enjoyable than rereading | 4% | 10% | 9% | 17% |
|  | I usually do not quiz myself | 9% | 9% | 12% | 4% |
| 7. Imagine that in the course of studying, you become convinced that you know the answer to a certain question. What would you do? | Make sure to study (or test yourself on) it again later | 36% | 46% | 38% | 43% |
|  | Put it aside and focus on other material | 64% | 54% | 62% | 56% |
| 8. What time of day do you most often do your studying? | Morning | - | <1% | 4% | 7% |
|  | Afternoon | - | 11% | 20% | 19% |
|  | Evening | - | 69% | 57% | 49% |
|  | Late night | - | 20% | 18% | 25% |
| 9. During what time of day do you believe your studying is (or would be) most effective? | Morning | - | 15% | 17% | 23% |
|  | Afternoon | - | 27% | 36% | 31% |
|  | Evening | - | 50% | 40% | 35% |
|  | Late night | - | 9% | 6% | 12% |
| 10. Which of the following best describes your pattern of study? | I most often space out my study sessions over multiple days/weeks | - | 47% | 48% | 51% |
|  | I most often do my studying in one session before the test | - | 53% | 52% | 48% |
| 11. Imagine your normal study routine. What, if any, other activities do you typically do while you’re studying? *(Please check all that apply* | Listen to music | - | - | - | 60% |
|  | Watch videos | - | - | - | 16% |
|  | Communicate using your phone | - | - | - | 42% |
|  | Browse social media | - | - | - | 26% |
|  | Browse the web generally | - | - | - | 8% |
|  | Talk to other people in person | - | - | - | 34% |
|  | None of the above | - | - | - | 16% |
| 12. Do you think it is more effective to focus only on what you’re studying, or to multitask while you study? | It is more effective to focus only on studying | - | - | - | 90% |
|  | It is more effective to multitask while studying | - | - | - | 8% |
| 13. What is your current grade point average? | 0.0-1.6 | - | 0% | 1% | 0% |
|  | 1.7 - 2.1 | - | 7% | 2% | <1% |
|  | 2.2 - 2.6 | - | 17% | 6% | 4% |
|  | 2.7 - 3.1 | - | 24% | 22% | 14% |
|  | 3.2 - 3.6 | - | 36% | 38% | 26% |
|  | 3.7 - 4.0 | - | 17% | 31% | 19% |
|  | This is my first semester in college, so I don't have a college GPA yet | - | - | - | 32% |
|  | I don't know | - | - | - | 4% |
| 14. Which of the following study strategies do you use regularly? *(Please check all that apply)* | Test yourself with questions or practice problems | - | 71% | 72% | 75% |
|  | Use flashcards | - | 62% | 54% | 52% |
|  | Recopy your notes |  | 33% | 33% | 43% |
|  | Reread chapters, articles, notes, lecture slides, etc | - | 66% | 67% | 66% |
|  | Make outlines | - | 22% | 53% | 43% |
|  | Consult online resources that were provided by the instructor | - | - | - | 51% |
|  | Consult online resources that were NOT provided by your instructor | - | - | - | 44% |
|  | Underline or highlight while reading | - | 72% | - | 52% |
|  | Make diagrams, charts or pictures | - | 15% | 24% | 25% |
|  | Study with friends | - | 50% | 48% | 51% |
|  | Use a mnemonic technique | - | - | - | 42% |
|  | Cram information the night before the test | - | 66% | 53% | 51% |
|  | Ask questions or verbally participate during class | - | 37% | 25% | 37% |
|  | Other | - | 6% | 4% | - |

Table S2

*Response Percentages Pre and Post Covid-19*

| Questions | Responses | Pre-Covid | Post-Covid | Effect size (*V_C_)* |
| --- | --- | --- | --- | --- |
| 1.Would you study the way you do because a teacher (or teachers) taught you to study that way? | Yes | 56% | 50% | .06 |
|  | No | 43% | 49% |  |
| 2. How do you decide what to study next? | Whatever's due soonest/overdue | 62% | 62% | .10 |
|  | Whatever I haven't studied for the longest time | 2% | 1% |  |
|  | Whatever I find interesting. | 9% | 6% |  |
|  | Whatever I feel I'm doing the worst in | 10% | 8% |  |
|  | I plan my study schedule ahead of time, and I study whatever I've scheduled. | 18% | 24% |  |
| 3. Do you usually return to course material to review it after a course has ended? | Yes | 24% | 23% | <.001 |
|  | No | 76% | 76% |  |
| 4. All other things being equal, what do you study more for? | Essay/short answer exams | 21% | 29% | .11 |
|  | Multiple choice exams | 18% | 21% |  |
|  | About the same | 61% | 50% |  |
| 5. When you study, do you typically read a textbook/article more than once? | Yes, I reread whole chapter/articles | 11% | 10% | .08 |
|  | I reread sections that I underlined highlighted or marked | 58% | 64% |  |
|  | Not usually | 31% | 26% |  |
| 6. If you quiz yourself while you study (either using a quiz at the end of a chapter, or a practice quiz, or flashcards, or something else) why do you do it? | I learn more that way that I would through rereading | 37% | 29% | .08 |
|  | To figure out how well I have learned the information I'm studying | 44% | 50% |  |
|  | I find quizzing more enjoyable than rereading | 15% | 17% |  |
|  | I usually do not quiz myself | 4% | 5% |  |
| 7. Imagine that in the course of studying, you become convinced that you know the answer to a certain question. What would you do? | Make sure to study (or test yourself on) it again later | 44% | 43% | 0 |
|  | Put it aside and focus on other material | 56% | 56% |  |
| 8. What time of day do you most often do your studying? | Morning | 4% | 8% | .09 |
|  | Afternoon | 18% | 20% |  |
|  | Evening | 51% | 49% |  |
|  | Late night | 27% | 23% |  |
| 9. During what time of day do you believe your studying is (or would be) most effective? | Morning | 21% | 24% | .11 |
|  | Afternoon | 30% | 32% |  |
|  | Evening | 41% | 31% |  |
|  | Late night | 8% | 12% |  |
| 10. Which of the following best describes your pattern of study? | I most often space out my study sessions over multiple days/weeks | 56% | 50% | .05 |
|  | I most often do my studying in one session before the test | 44% | 49% |  |
| 11. Imagine your normal study routine. What, if any, other activities do you typically do while you’re studying? *(Please check all that apply* | Listen to music | 63% | 58% | .07 |
|  | Watch videos | 22% | 14% |  |
|  | Communicate using your phone | 43% | 42% |  |
|  | Browse social media | 27% | 26% |  |
|  | Browse the web generally | 11% | 9% |  |
|  | Talk to other people in person | 46% | 33% |  |
|  | None of the above | 15% | 17% |  |
| 12. Do you think it is more effective to focus only on what you’re studying, or to multitask while you study? | It is more effective to focus only on studying | 93% | 91% | .01 |
|  | It is more effective to multitask while studying | 7% | 8% |  |
| 13. What is your current grade point average? | 0.0-1.6 | 0% | 0% | .20* |
|  | 1.7 - 2.1 | 2% | 0% |  |
|  | 2.2 - 2.6 | 3% | 5% |  |
|  | 2.7 - 3.1 | 15% | 17% |  |
|  | 3.2 - 3.6 | 29% | 26% |  |
|  | 3.7 - 4.0 | 11% | 25% |  |
|  | This is my first semester in college, so I don't have a college GPA yet | 37% | 25% |  |
|  | I don't know | 3% | 3% |  |
| 14. Which of the following study strategies do you use regularly? *(Please check all that apply)* | Test yourself with questions or practice problems | 73% | 77% | .06 |
|  | Use flashcards | 60% | 50% |  |
|  | Recopy your notes | 44% | 42% |  |
|  | Reread chapters, articles, notes, lecture slides, etc. | 57% | 69% |  |
|  | Make outlines | 47% | 43% |  |
|  | Consult online resources that were provided by the instructor | 48% | 53% |  |
|  | Consult online resources that were NOT provided by your instructor | 43% | 46% |  |
|  | Underline or highlight while reading | 49% | 55% |  |
|  | Make diagrams, charts or pictures | 30% | 22% |  |
|  | Study with friends | 58% | 50% |  |
|  | Use a mnemonic technique | 50% | 40% |  |
|  | Cram information the night before the test | 50% | 51% |  |
|  | Ask questions or verbally participate during class | 40% | 38% |  |
